# Oleocanthal Protects C2C12 Myotubes against the Pro-Catabolic and Anti-Myogenic Action of Stimuli Able to Induce Muscle Wasting *In Vivo*

**DOI:** 10.3390/nu16091302

**Published:** 2024-04-26

**Authors:** Daniela De Stefanis, Andrea Balestrini, Paola Costelli

**Affiliations:** Department of Clinical and Biological Sciences, University of Turin, 10125 Turin, Italy; daniela.destefanis@unito.it (D.D.S.); andre.bale96@gmail.com (A.B.)

**Keywords:** muscle wasting, TNF-α, cachexia, tumor-conditioned medium, myogenesis

## Abstract

Oleocanthal (OC) is a monophenol of extra-virgin olive oil (EVOO) endowed with antibiotic, cardioprotective and anticancer effects, among others, mainly in view of its antioxidant and anti-inflammatory properties. OC has been largely investigated in terms of its anticancer activity, in Alzheimer disease and in collagen-induced arthritis; however, the possibility that it can also affect muscle biology has been totally overlooked so far. This study is the first to describe that OC modulates alterations induced in C2C12 myotubes by stimuli known to induce muscle wasting in vivo, namely TNF-α, or in the medium conditioned by the C26 cachexia-inducing tumor (CM-C26). C2C12 myotubes were exposed to CM-C26 or TNF-α in the presence or absence of OC for 24 and 48 h and analyzed by immunofluorescence and Western blotting. In combination with TNF-α or CM-C26, OC was revealed to be able to restore both the myotube’s original size and morphology and normal levels of both atrogin-1 and MuRF1. OC seems unable to impinge on the autophagic–lysosomal proteolytic system or protein synthesis. Modulations towards normal levels of the expression of molecules involved in myogenesis, such as Pax7, myogenin and MyHC, were also observed in the myotube cultures exposed to OC and TNF-α or CM-C26. In conclusion, the data presented here show that OC exerts a protective action in C2C12 myotubes exposed to TNF-α or CM-C26, with mechanisms likely involving the downregulation of ubiquitin–proteasome-dependent proteolysis and the partial relief of myogenic differentiation impairment.

## 1. Introduction

Extra-virgin olive oil (EVOO), the main source of fat in the Mediterranean diet, contains several bioactive molecules, among which are phenols such as tyrosol, hydroxytyrosol, oleacein, ligstroside aglycone and oleocanthal (OC). They appear to exert antibiotic, cardioprotective, and anticancer effects, among others, mainly in view of their antioxidant and anti-inflammatory properties [1,2].

OC is a monophenol belonging to the category of secoiridoids which has been largely investigated in terms of its anticancer activity [3]. Indeed, data obtained using different cancer cell lines have shown that it impinges on the number of cells by inhibiting proliferation and inducing apoptosis [4]. Similar observations have been reported in experimental models of liver, breast and lung cancer [5]. Another study reported that an EVOO extract enriched with OC could potentiate the cytotoxic action of the pro-inflammatory cytokine TNFα on liver cancer cell lines [6]. In addition, OC was also reported to exert beneficial effects in experimental models of Alzheimer disease [7] and collagen-induced arthritis [8]. Up to now, there have been very few clinical trials aimed at investigating the beneficial effects of OC in human beings [5,9].

Polyphenols such as resveratrol, curcumin and epigallocatechin-3-gallate (EGCG) have been proposed to positively act on muscle homeostasis, particularly when it is altered by stimuli such as diet, hormones or diseases. EVOO-derived phenols have been poorly investigated in terms of their potential benefit on the skeletal muscle system. Generally speaking, they have been shown to protect against aging-related mitochondrial dysfunction by enhancing intracellular antioxidant defenses. Consistently, they have also been shown to delay cellular senescence by protecting against inflammaging [10]. With a loss of muscle mass and function being one of the hallmarks of aging, it is likely that EVOO-derived phenols might exert beneficial effects at the muscle level as well. However, the data available in the literature are too scanty to provide a clear-cut answer to this issue. To begin with, very few studies have addressed the relevance of regularly consuming EVOO in the onset and progression of sarcopenia [10], while at present, there are no reports on the effects of specific EVOO-derived phenols. Little information in this regard is available in conditions characterized by muscle atrophy that differs from aging-related sarcopenia. Indeed, hydroxytyrosol was reported to prevent the impairment of muscle mitochondria induced in rats by a high-fat diet or by strenuous exercise [11,12], while, when used in combination with other compounds, it was shown to mimic a reloading effect in a model of disuse-induced muscle atrophy [13]. Hydroxytyrosol acetate was shown to preserve mitochondrial membrane potential and to prevent myotube death in cultures exposed to oxidative stress conditions. It was also able to enhance both the mitochondrial respiration capacity and activity of mitochondrial complexes I and II [14]. Oleuropein revealed ROS scavenging action in C2C12 cultures [15] and was shown to reduce mitochondrial ROS production in chicken muscle primary cultures by activating sirtuin 1, resulting in an increased expression of the master regulator of mitochondrial biogenesis, PGC-1α [16]. At present, there is just one study reporting the potential use of tyrosol to protect skeletal muscle cells against oxidative stress induced by hyperglycemia [17].

While OC has been widely investigated as an anticancer agent, the possibility that it could also impinge on muscle biology has been totally neglected up until now. In this regard, the present study is the first to investigate the effects of OC on myotube cultures exposed to stimuli able to mimic a pathological muscle microenvironment, namely the pro-inflammatory cytokine TNF-α or the medium conditioned by a cachexia-inducing experimental tumor [18].

## 2. Materials and Methods

### 2.1. Reagents for the Experiments

All reagents, unless otherwise indicated, were purchased from Sigma Aldrich, Milan, Italy.

### 2.2. Cell Lines

#### 2.2.1. C2C12

Murine C2C12 skeletal myoblasts (ATCC, Manassas, VA, USA) were grown in high-glucose DMEM supplemented with 10% fetal bovine serum (FBS), 100 U/mL of penicillin, 100 mg/mL of streptomycin, 100 mg/mL of sodium pyruvate and 2 mM of L-glutamine (growth medium; GM) and maintained at 37 °C in a humidified atmosphere of 5% CO_2_ in air. Myoblasts were seeded on 12-well plates at a density of 30,000/cm^2^ in GM. To induce differentiation, after 24 h (sub-confluency), GM was replaced by DMEM containing 100 U/mL of penicillin, 100 mg/mL of streptomycin, 100 mg/mL of sodium pyruvate and 2 mM of L-glutamine, supplemented with 2% horse serum (differentiation medium; DM). The medium was changed every 48 h. After 5 days of culture in DM, mature myotubes aligned to form regular bundles. 

#### 2.2.2. C26 

C26 cells [obtained in 2005 from Prof M.P. Colombo (National Cancer Institute, Milano, Italy)] were maintained in vitro in DMEM supplemented with 10% FBS, 100 U/mL of penicillin, 100 mg/mL of streptomycin, 100 mg/mL of sodium pyruvate and 2 mM of L-glutamine at 37 °C in a humidified atmosphere of 5% CO_2_ in air. To prepare the conditioned medium (CM-C26), C26 cells were plated at a density of 8000 cells/cm^2^. Upon reaching about 90% confluence, the C26 monolayer was washed twice with PBS, and myotube DM was added, as indicated in a protocol by Jackman et al. [19]. After 24 h, the CM-C26 was collected and centrifuged at 800 rpm for 10 min at 4 °C (ALC centrifuge mod. 4227R) to remove any dead cells and cell debris. The supernatant was sterilized by passing it through a sterile filter with 0.2 μm diameter pores. For C2C12 cell experiments, CM-C26 was diluted in a ratio of 1:3 with DM and used for the experiments as indicated below.

### 2.3. MTT Assay

Cell viability analysis with an 3-(4,5-Di-2-yl)-2,5-ditetrazolium bromide (MTT) assay was performed to evaluate the cytotoxic effect of OC. OC was purchased from PhytoLab, GmbH & Co, Vestenbergsgreuth, Germany (product code: 83882; purity: 97%). The cells were seeded in 96-well plates and differentiated as previously described. On the fifth day of C2C12 myotube differentiation, the cells (8 wells per condition) were treated with different concentrations of OC (1–20 μM) for the following 24 or 48 h. At the end of the incubation period, the cells were incubated with 20 µL of MTT solution (5 mg/mL). Three hours later, the absorbance was measured at 570 nm, and the data were assessed using an iMark Microplate Reader (Bio-Rad Laboratories, Segrate, Italy). Based on the results in Figure S1, a 10 µM concentration was chosen.

### 2.4. Treatments

For the experiments, the cells were seeded in 12-well plates (2 wells per condition) and differentiated as previously described. On the fifth day of C2C12 myotube differentiation, the medium was removed, and, after monolayer washing, the cultures were exposed for a further 24 or 48 h to the following treatments:

- Control (Co): DM;
- CM-C26: diluted CM-C26 (1:3 in DM);
- TNF-α: DM containing 100 ng/mL of TNF-α;
- OC: DM containing 10 µM of OC;
- CM-C26 + OC: diluted CM-C26 + 10 µM of OC.
- TNF + OC: DM + TNF-α and OC with final concentrations of 100 ng/mL and 10 µM, respectively.

The medium was changed every 24 h.

### 2.5. Immunofluorescence

Myotubes were grown and treated as described above. After 24 or 48 h, cells were washed with PBS, fixed for 10 min in 4% PFA, then blocked with 0.1% Triton X-100 and 1% BSA in PBS. Fixed monolayers were incubated with primary (anti-MyHC and Ki67; Invitrogen, Carlsbad, CA, USA) and secondary antibodies (Alexa-555, Alexa-488, respectively; Invitrogen, USA). To counterstain the nuclei, cells were incubated with Hoechst 33342 for 10 min. Photographs were taken with an inverted-phase contrast microscope (Zeiss, Oberkochen, Germany, Axiovert 35), and images were processed with ImageJ version 1.53c.

### 2.6. Protein Synthesis

To evaluate the relative rate of protein synthesis, the SUnSET methodology was used [20]. First, 1 μM of puromycin was added to C2C12 myotube culture medium 30’ before monolayer collection. Cells were then lysed, and the amount of puromycin incorporated into nascent peptides was detected by Western blotting using a specific anti-puromycin antibody as described below.

### 2.7. Western Blotting

Cells were lysed in RIPA buffer (1% NP40, 0.5% NaDOC, 0.1% SDS) supplemented with 1 mM of DTT, 0.1 mM of PMSF, 2 mg/mL of aprotinin, 2 mg/mL of leupeptin and 100 mM of NaF. Protein content was determined using a Bio-Rad Protein Assay Kit (Bio-Rad, Hercules, CA, USA). Equal amounts of protein (50 μg) were fractionated on polyacrilamide SDS gel and transferred to a nitrocellulose membrane (BioRad, Hercules, CA, USA). The membranes were blocked with a solution containing 5% non-fat milk (or 5% BSA for phosphorylated protein) in TBS 0.05% Tween and incubated with primary antibodies overnight at 4 °C. Subsequently, the membranes were incubated with a secondary antibody coupled with horseradish peroxidase (Bio-Rad, Hercules, CA, USA). Reactive proteins were visualized using an ECL Western Blotting Detection kit (Amersham Biosciences, UK) and analyzed through the ChemiDoc image analysis system (BioRad, Hercules, CA, USA). The results obtained were normalized as indicated in the figure legend.

### 2.8. Statistical Analysis

All values are expressed as the mean ± SEM. The significance among mean values was evaluated by applying a two-way analysis of variance (ANOVA) followed by a Bonferroni post hoc test using RStudio 4.3.1 software. A *p* value < 0.05 was considered statistically significant.

## 3. Results

### 3.1. OC Partially Restores Myotube Morphology 

**Figure 1:**
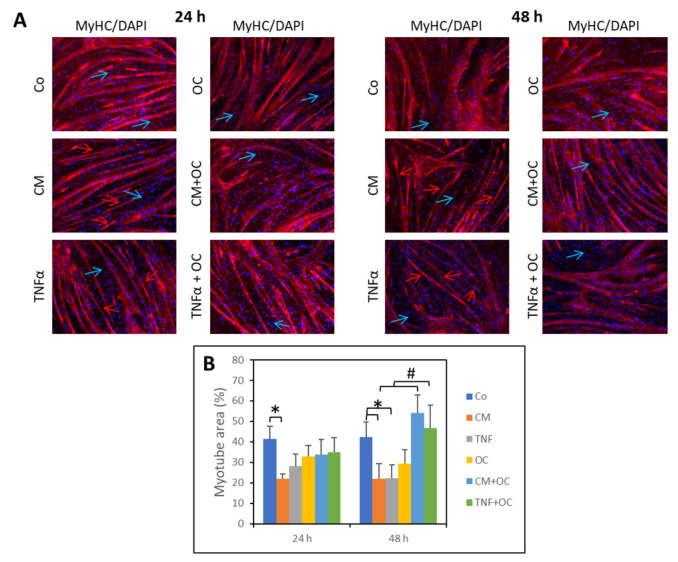
Effects of OC, CM-C26 or TNF-α on myotube morphology. (**A**) MyHC expression (immunofluorescence) in myotubes exposed to CM-C26 or TNF-α in the presence or absence of OC for 24 and 48 h. Nuclei are stained with DAPI. Light blue arrows: undifferentiated myoblasts; red arrows: myotubes with length and diameter lower than controls (Co); (**B**) myotube area quantification. Magnification: 10×. The results are representative of three independent experiments, and each experimental condition is duplicated. Data are expressed as means ± SEM. Significance of the differences: *—*p* < 0.05 vs. Co; #—*p* < 0.05 vs. CM-C26 or TNF-α.

The myotube area was assessed in 5-day-differentiated C2C12 cultures in the presence or absence of CM-C26 or TNF-α for 24 and 48 h (Figure 1A,B). The control cultures (Co) showed myotubes with a regular diameter and elongated and uniform morphology; as expected, undifferentiated myoblasts (negative for MyHC staining) were still present. In contrast, the myotube area appeared to be reduced in the cultures exposed to CM-C26 (−47%) for 24 h. A similar, though not significant, trend toward reduction was induced by TNF-α (−33%). Exposure to OC alone did not significantly change the myotube morphology, with only a slight diameter reduction occurring in a few myotubes. When the myocyte cultures were simultaneously exposed to OC and CM-C26 or TNF-α, the myotube diameter, length and area were improved in comparison to the cultures treated with CM-C26 or TNF-α alone (Figure 1A,B).

After 48 h, treatment with CM-C26 or TNF-α reduced the myotube area. Such an effect was partially prevented by the presence of OC in the culture medium (Figure 1A,B), which resulted in a marked increase in the myotube surface area (+146% and +109% in the CM-C26 + OC- and TNF-α + OC-treated cultures, respectively).

### 3.2. Molecular Markers of Protein Catabolism Are DownRegulated by OC

**Figure 2:**
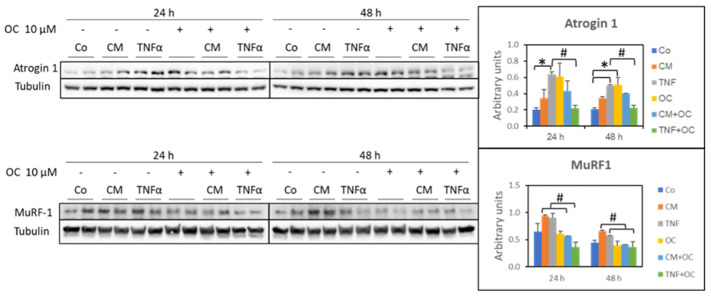
Effects of OC, CM-C26 or TNF-α on atrogin-1 and MuRF-1 expression. The results are representative of three experiments, and each experimental condition is duplicated. Data are expressed as means ± SEM. Significance of the differences: *—*p* < 0.05 vs. Co; #—*p* < 0.05 vs. CM-C26 or TNF-α.

Atrogin-1 and MuRF-1 are two muscle-specific ubiquitin ligases whose increased expression is often used as an index of protein hypercatabolism due to the ubiquitin–proteasome proteolytic system. Conforming to previous findings [21,22], atrogin-1 was overexpressed in the myotubes cultured in the presence of TNF-α (24 and 48 h), while it was not significantly affected by exposure to CM-C26. The addition of OC effectively downregulated TNF-α-induced atrogin-1 overexpression. Of interest, OC alone resulted in increased atrogin-1 protein levels (Figure 2). 

As for MuRF-1 protein expression, the myotubes exposed to CM-C26 or TNF-α showed an increasing trend, which was restored to the Co values when OC was also present in the culture medium at both 24 and 48 h (Figure 2).

Given the upregulation of atrogin-1 and MuRF-1, the expression levels of ubiquitylated proteins were analyzed, showing that they were not modified in myotubes exposed to CM-C26 or TNF-α. However, when OC was present in the culture medium, either alone or in association with CM-C26, though not with TNF-α, ubiquitylated proteins were significantly reduced in comparison to the Co levels or CM-C26, respectively (Figure S2). After 48 h, on the other hand, none of the experimental conditions resulted in significant changes in the levels of ubiquitylated proteins (Figure S2).

In addition to the ubiquitin–proteasome system, intracellular protein degradation relies on autophagic–lysosomal proteolysis activity. To investigate the relevance of the latter to the catabolic drift likely induced in myotubes by TNF-α or CM-C26, the expression of molecular markers of autophagy, namely beclin-1, LC3-B and p62/SQTM1, was assessed. On the whole, the results did not reveal important changes in the present experimental setting, despite trends suggesting that the system could be overactivated by the treatments and partially restored to normal levels by the addition of OC, particularly after 24 h (Figure S3). In contrast, after 48 h, OC alone or in the presence of CM-C26 resulted in increased beclin-1 levels (Figure S3).

**Figure 3:**
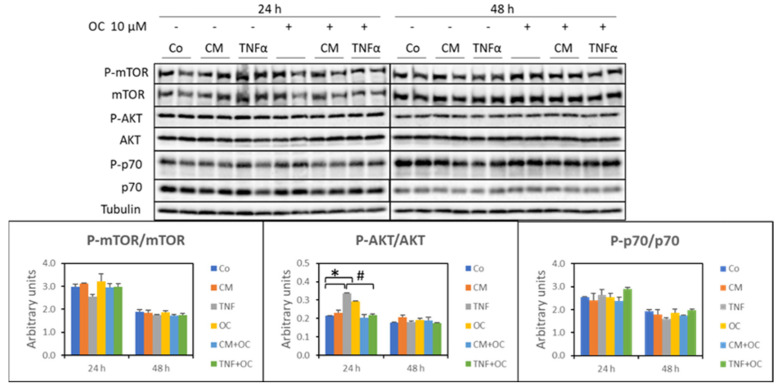
Molecular markers of protein anabolism in myotube cultures exposed to OC, CM-C26 or TNF-α for 24 and 48 h. The results are representative of three experiments, and each experimental condition is duplicated. Data are expressed as means ± SEM. Significance of the differences: *—*p* < 0.05 vs. Co; #—*p* < 0.05 vs. TNF-α.

Impaired protein synthesis could also have contributed to the reduced myotube diameter and length induced by TNF-α or CM-C26 (see above). This issue was investigated by assessing the expression levels of molecular markers of protein anabolism, such as the ratio between phosphorylated and total mTOR, Akt and p70, and by measuring the incorporation of puromycin into proteins, which gives an estimate of the protein synthesis rates [20]. The results, however, did not provide clear-cut evidence that protein anabolism was perturbed in the present experimental setting. Indeed, the p-mTOR/mTOR and p-p70/p70 ratios were unchanged upon exposure to TNF-α or CM-C26, irrespective of the presence of OC. A different pattern could be observed after 24 h only for the p-Akt/Akt ratio, which appeared increased in the presence of TNF-α or OC and reduced when the myotubes were exposed to the combination of OC + TNF-α (Figure 3).

**Figure 4:**
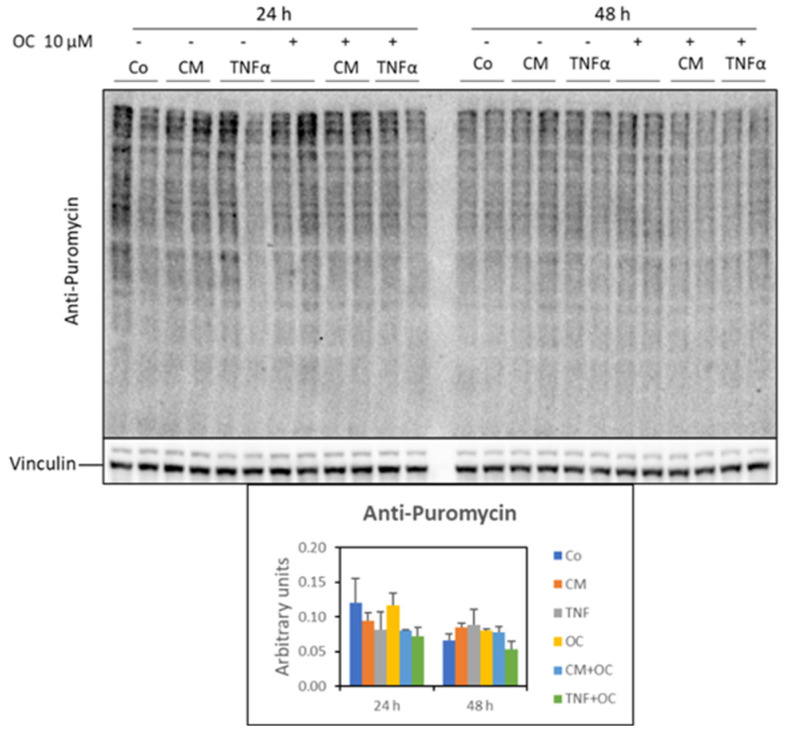
Analysis of protein neosynthesis in myotubes treated with OC, CM-C26 or TNF-α for 24 and 48 h. The results are representative of three experiments, and each experimental condition is duplicated. Data are expressed as means ± SEM.

As for protein synthesis rates, no significant change was observed in the myotubes exposed to TNF-α or CM-C26 in the presence or absence of OC (Figure 4).

### 3.3. Effects of OC on Myogenic Differentiation

**Figure 5:**
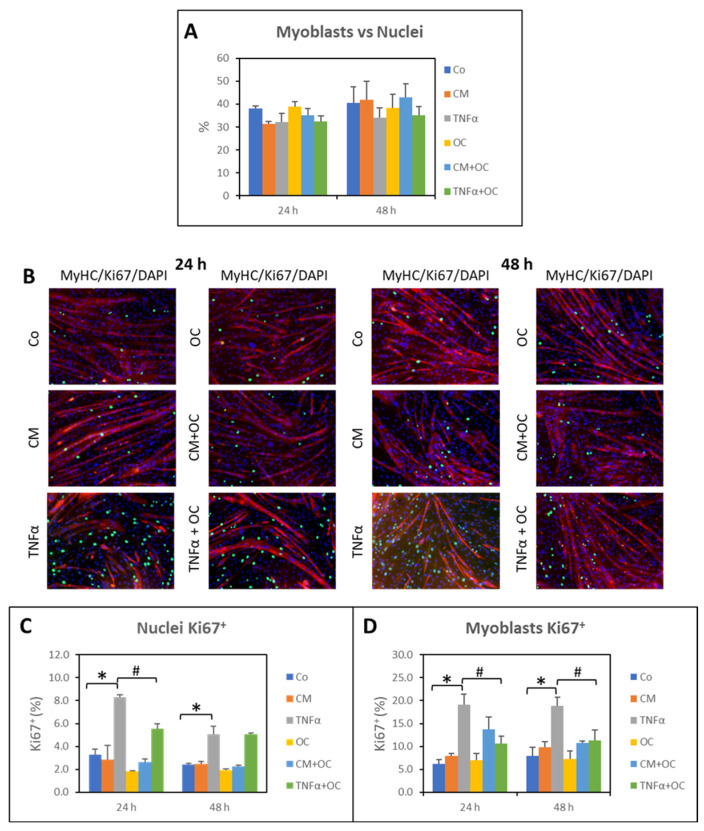
Ki67 expression is modulated by TNF-α and OC. (**A**) Quantification of myoblast number versus total nuclei. (**B**) Myotubes were cultured for 24 and 48 h in the presence of TNF-α or CM-C26 with or without OC. Cultures were stained with anti-Ki67 (green) and anti-MyHC (red) antibodies. Nuclei were counterstained with DAPI. Magnification: 10×. (**C**) Quantification of Ki67^+^ cells versus total nuclei. (**D**) Quantification of myoblasts Ki67^+^ versus total nuclei. The results are representative of three experiments, and each experimental condition is duplicated. Data are expressed as means ± SEM. Significance of the difference *—*p* < 0.05 vs. Co; #—*p* < 0.05 vs. TNF-α.

The morphological analysis showed the presence of many undifferentiated myoblasts in the myotube cultures (see Figure 1), although no changes could be seen among the different experimental conditions (Figure 5A). In order to understand if these myoblasts are quiescent or cycling, the expression of Ki67, a marker of cell proliferation, was evaluated. Figure 5B shows the presence of Ki67^+^ nuclei, all located in inter-myotube spaces and negative for MyHC, thus identifiable with myoblasts. Differently from the number of myoblasts, the number of Ki67^+^ nuclei was significantly modulated by the treatments. Indeed, after 24 and 48 h, TNF-α caused a marked increase in the percentage of Ki67^+^ cells (green staining), while the cultures exposed to CM-C26 or OC alone remained similar to the Co. Consistently, when TNF-α or CM-C26 was combined with OC, an effect could be observed in the OC + TNF-α condition only and just at the 24 h time point, when the percentage of Ki67^+^ cells was significantly reduced in comparison with the TNF-α-treated cultures. Such a modulation was lost in the 48 h cultures exposed to both OC and TNF-α (Figure 5C) but was still present when only Ki67^+^ myoblasts were counted (Figure 5D). 

**Figure 6:**
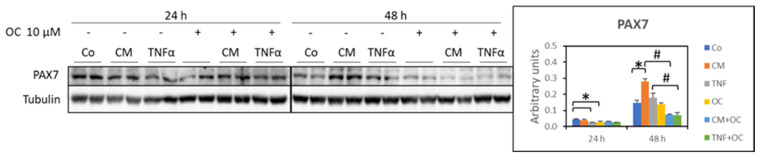
Pax7 expression in myotubes treated with OC, CM-C26 or TNF-α for 24 and 48 h. The results are representative of three experiments, and each experimental condition is duplicated. Data are expressed as means ± SEM. Significance of the differences: *—*p* < 0.05 vs. Co; #—*p* < 0.05 vs. CM-C26 or TNF-α.

Since the percentage of Ki67^+^ cells increased in the myotube cultures exposed to TNF-α and was partially reduced in the presence of both TNF-α and OC, the expression of Pax7, a marker of early myogenesis, was investigated. In the myotubes exposed to TNF-α or OC for 24 h, Pax7 expression was significantly reduced compared to the Co values (−43% and −44%, respectively), while no differences could be observed in the OC + CM-C26 or OC + TNF-α cultures. In contrast, Pax7 expression markedly increased in the myotubes exposed to CM-C26 for 48 h (+89% than Co), though not in those exposed to TNF-α. However, a significant reduction in Pax7 levels could be observed in the 48 h OC + TNF-α and OC + CM-C26 cultures (−74% and −60%, respectively; Figure 6).

**Figure 7:**
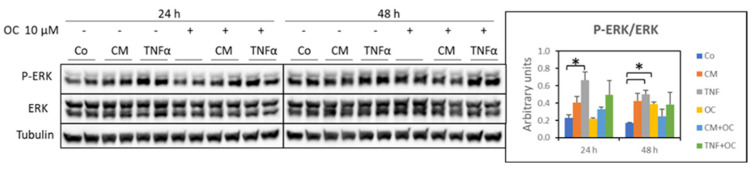
Expression of ERKs in myotube cultures treated with OC, CM-C26 or TNF-α for 24 and 48 h. The results are representative of three experiments, and each experimental condition is duplicated. Data are expressed as means ± SEM. Significance of the differences: *—*p* < 0.05 vs. Co.

The increased Pax7 levels in the myotubes exposed to CM-C26 for 48 h are consistent with a previous observation showing that Pax7 is overexpressed in the skeletal muscle of mice implanted with a C26 tumor [23,24]. Such overexpression was associated with high levels of phosphorylated extracellular signal-regulated kinases (ERKs). Along this line, pERK expression was assessed in the myotubes exposed to TNF-α or CM-C26 in the presence or absence of OC. The results reported in Figure 7 show that treatment with TNF-α for 24 h and 48 h significantly increased the pERK levels in comparison to the Co (+193% and +200%, respectively), confirming previous observations [23]. In contrast, exposure to CM-C26 did not result in significant changes in pERK expression, although an increasing trend could be seen in the 24 h cultures. OC exposure for 48 h resulted in increased pERK expression, while its combination with TNF-α or CM-C26 did not impinge on the pERK levels at any time point (Figure 7).

**Figure 8:**
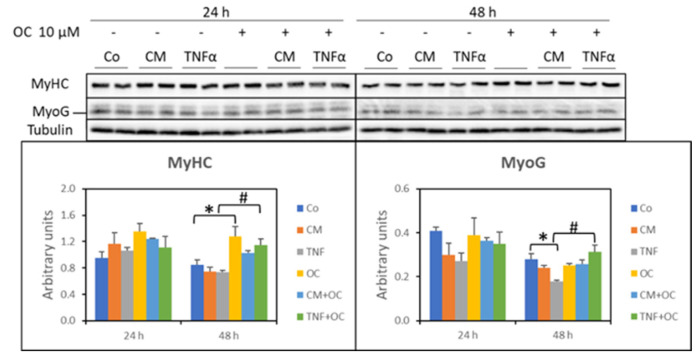
MyHC and MyoG protein expression in myotube cultures treated with OC, CM-C26 or TNF-α for 24 and 48 h. The results are representative of three experiments, and each experimental condition is duplicated. Data are expressed as means ± SEM. Significance of the differences: *—*p* < 0.05 vs. Co; #—*p* < 0.05 vs. TNF-α.

Finally, the expression of the following markers of differentiation was assessed: (i) MyHC, as an indicator of the stage of myogenesis, and (ii) MyoG, which allows myoblast maturation into myocytes, driving the formation of myotubes/myofibers [25]. In the present study, both CM-C26 and TNF-α were unable to impinge on MyHC expression in the C2C12 myotube cultures. While this pattern was not modified by the addition of OC after 24 h, after 48 h, the MyHC levels increased in the presence of OC or OC + TNF-α compared to the Co or TNF-α, respectively (Figure 8). As for MyoG levels, they were significantly reduced by TNF-α at a 48 h treatment time, while only a decreasing trend could be observed for CM-C26. The addition of OC to the culture medium resulted in restored MyoG expression in the 48 h TNF-α-treated myotubes (75% more than in those with TNF-α alone; Figure 8). 

## 4. Discussion

This study is the first to describe the ability of OC to modulate alterations induced in C2C12 myotubes by stimuli known to induce muscle wasting in vivo.

In order to mimic a pathological muscle microenvironment, C2C12 myotubes were exposed to the pro-inflammatory cytokine TNF-α or to a medium conditioned by C26 cells (CM-C26), a well-characterized tumor known for its ability to cause cachexia when implanted into a host mouse [19]. In accordance with the data available in the literature [23,26], both stimuli result in a reduction in the size of the myotubes, associated with an increased expression of the muscle-specific ubiquitin ligases atrogin-1 and MuRF1. When TNF-α or CM-C26 are associated with OC, a protective effect can be observed on the myotubes, whose size is restored to the control levels after a 48 h culture. This result cannot be compared with data reported in the literature since no studies investigating the action of OC or other EVOO-derived polyphenols on myotube size are available. In this regard, however, Wang and collaborators [14] showed that the oxidative stress-induced degeneration of myotube morphology could be prevented by hydroxytyrosol acetate, supporting the view that EVOO-derived polyphenols can impinge on cultured myotubes. On the other hand, polyphenols not present in EVOO, such as resveratrol and curcumin, have been shown to counteract the alterations in myotube morphology induced by glucose restriction or dexamethasone [26,27].

Myotube exposure to OC in combination with TNF-α or CM-C26 was revealed to be able to restore normal levels of both atrogin-1 and MuRF1, suggesting that the downregulation of proteasome-dependent proteolysis likely contributes to the OC-induced restoration of myotube size. The reduced expression of muscle-specific ubiquitin ligases caused by OC is consistent with previous results obtained from administering polyphenols such as EGCG, resveratrol, oligonol (a polyphenol derived from lychee), or curcumin to experimental animals to model muscle wasting associated with diabetes, denervation, aging or exercise-induced muscle damage [28,29,30,31]. Similarly, in C2C12 cultures, EGCG has been reported to prevent dexamethasone-induced MuRF1 overexpression [32], while oligonol restores normal levels of atrogin-1 and MuRF1 gene expression in myotubes exposed to palmitate [30]. As for EVOO-specific polyphenols, indirect hints come from a study showing that a mixture containing algae oil, EVOO and olive leaf extract effectively prevents atrogin-1 overexpression in rats with sepsis [33]. A more direct observation is provided by another report demonstrating that hydroxytyrosol prevents exercise-induced increases in both muscle atrogin-1 and MuRF1 mRNA levels [11]. 

While inhibiting the atrogin-1 and MuRF1 overexpression induced by TNF-α and CM-C26, OC seems unable to affect the autophagic–lysosomal proteolytic system, at least in the experimental setting used in the present study, limiting the analysis to the markers assessed here. This observation is not consistent with data reported in the literature, which show that polyphenols, including hydroxytyrosol, generally impinge on autophagy [11,34]. However, the results presented here suffer from the following limitations: (i) both TNF-α and CM-C26 do not significantly modulate beclin-1, LC3 and p62/SQSTM1 expression, suggesting that the absence of an altered autophagic steady state could nullify any OC effect, and (ii) a flux experiment has not been performed, and the possibility that changes in the expression of the three markers evaluated could have occurred on a time schedule different from that adopted in the present study cannot be discarded. On the whole, whether OC is effectively endowed with the ability to act on the autophagic–lysosomal proteolytic system still remains to be elucidated, paving the way for further investigations. 

The reduced myotube size shown in the present study is not associated with changes in protein synthesis or in the expression of related molecular markers (pAkt/Akt, pmTOR-mTOR, pP70S6K/P70S6K), with the increased pAkt/Akt ratio induced by TNF-α being the only modification in comparison to the control cultures. These observations do not conform to data reported in the literature, showing that both TNF-α and CM-C26 negatively impinge on anabolism, at least at the molecular level, and that polyphenols, hydroxytyrosol included, counteract such a negative drift [35]. As an example, differentiating C2C12 myocytes exposed to CM-C26 led to reduced levels of pAkt/Akt, pmTOR/mTOR and pP70S6K/P70S6K, which were restored to control values through the addition of a magnolol culture medium, a lignane derived from Magnolia officinalis [36]. Another report has shown that the protection exerted by resveratrol against TNF-α-induced myotube thinning is associated with increased pAkt/Akt, pmTOR-mTOR and pP70S6K/P70S6K ratios [37]. Partially, at least, the discrepancy between the data in the literature and those reported here could depend on the different experimental schedules adopted. However, in none of the studies investigating the effects of polyphenols on myotubes exposed to TNF-α or CM-C26 has protein synthesis been assessed in association with the anabolism-related molecular profile. The latter, indeed, could be poised towards an activated setting at the molecular level but not necessarily result in protein accretion. The data shown in the present study suggest that OC does not impinge on myotube protein synthesis, highlighting the need for further investigations to clarify this issue.

Myogenic differentiation is well known to be inhibited in the presence of TNF-α [23] or CM-C26 [37]. Similarly, delayed muscle regeneration occurs in the muscles of C26-bearing mice and has been proposed to contribute to cancer-induced muscle wasting [23,24,38]. C2C12 myotube cultures are well known to maintain a small population of undifferentiated, quiescent cells, characterized by high Pax7, low myogenin and low Ki67 expression [39]. The latter is generally used to identify proliferating cells, being expressed during all phases of the cell cycle but not in G0 [40]. Despite all the experimental conditions having the same number of undifferentiated cells, many of these latter were Ki67^+^ in the cultures exposed to TNF-α, while no changes could be observed when comparing the control and CM-C26-treated myotubes. This observation demonstrates that some of the residual myoblasts in the myotube cultures still retained the ability to proliferate in the presence of adequate stimuli, TNF-α in particular. Since the cytokine was added to cultures 5 days after shifting to DM, the increased number of Ki67^+^ myoblasts cannot be due to the inhibition of myotube formation. Of interest, OC reduces Ki67^+^ cells in myotube cultures exposed to TNF-α, an observation which is consistent with the improved myotube size (reported in Figure 1), with the reduced expression of Pax7 (Figure 6) and with the increased expression of myogenin (Figure 8), demonstrating that this EVOO polyphenol can reduce, partially at least, the inhibition of myogenic differentiation induced by TNF-α.

The modulations of Pax7 expression reported here do not contrast with the hypothesis above. Indeed, in agreement with previous studies, Pax7 was reduced (24 h) or unchanged (48 h) in the cultures exposed to TNF-α [23], while it was markedly increased in the myotubes treated with CM-C26 for 48 h, likely paralleling Pax7 overexpression in the skeletal muscle of the C26 hosts [23,24]. These observations are consistent with the proliferative state of residual myoblasts induced by TNF-α but not those induced by CM-C26. Of interest, Pax7 expression was markedly reduced below the control values by OC + TNF-α and OC + CM-C26 in the 48 h cultures. Decreased Pax7 expression is a typical feature of myogenesis, and at present, very few studies have shown that polyphenols can impinge on this process. C2C12 myotube formation has been shown to be improved by resveratrol [41] or by EGCG, the latter being able to activate MyoD transcription by enhancing TAZ nuclear localization [42]. Moreover, rosmarinic acid has been reported to counteract the negative impact of heat stress on both myoblast apoptosis and differentiation into myotubes [43]. Along these lines, this study reports for the first time the possibility that OC could be relevant in reversing inflammation-driven impairment of myogenic differentiation. Consistently, the MyoG and MyHC levels were higher in the cultures treated with OC + TNF-α than those treated with TNF-α alone, paralleling the decreased Pax7 expression and the reduced number of Ki67^+^ cells. In contrast, despite being effective in reducing Pax7 expression, OC was revealed to be unable to modify both the MyoG and MyHC levels in the myotubes exposed to CM-C26, suggesting that the myogenic impairment induced by the C26-derived secretome relies on different mechanisms, partially at least, from those activated by TNF-α. In this regard, previous studies have shown that C26-derived extracellular vesicles, likely due to their microRNA content, miR-148a-3p and miR-181a-5p in particular, negatively affect the differentiation of C2C12 myoblasts [44]. Whether those microRNAs are relevant to maintaining residual myoblasts in the Pax7^+^ state and whether their action can be modulated by OC is currently unknown. In this regard, however, OC has been shown to reduce the hepatic expression of miR-181-5p in an experimental model of liver fibrosis [45]. Finally, the authors acknowledge that investigating the effect of OC on myoblast cultures exposed to TNF-α or CM-C26 would have improved the consistency of the results reported here. 

Previous observations have shown that C26-induced muscle wasting as well as the altered myotube formation occurring in TNF-α-treated C2C12 cultures can be reverted, partially at least, by ERK inhibition [23]. Along this line, the present data show that the pERK/ERK ratio was increased in the myotube cultures exposed to TNF-α, while only an increasing trend could be observed in the presence of CM-C26. Such a pattern was not modified by the combination of TNF-α or CM-C26 with OC, likely ruling out an effect on ERK-dependent signaling as the driver of OC’s protective activity. While the experimental schedule could be relevant to such an apparent lack of effect, it must be underlined that data showing the effects of polyphenols on ERK activation are scanty and contradictory, reporting an induction by resveratrol [41] but an inhibition by quercetin [46], EGCG [47] or nobiletin [48], and to the best of our knowledge, there are no studies referring to EVOO-derived polyphenols.

## 5. Conclusions

On the whole, the data presented here show that OC is able to protect C2C12 myotubes from a reduction in size due to exposure to TNF-α or to CM-C26, with mechanisms likely involving the downregulation of ubiquitin–proteasome-dependent proteolysis and the partial relief of myogenic differentiation impairment. While several issues discussed above still remain to be clarified, the present data demonstrate that OC can impinge on myotube culture homeostasis and open the way to the exploitation of EVOO-derived polyphenols as compounds able to positively interfere with the muscle wasting featured in several chronic diseases.

## Data Availability

All data that support the findings of this study are available within the article or Supplementary Material.

## Supplementary Materials

The following supporting information can be downloaded at https://www.mdpi.com/article/10.3390/nu16091302/s1: Figure S1. MTT assay on C2C12 myotube cultures exposed for 24 and 48 h to different OC concentrations. The results are representative of three experiments, with *n* = 8 for each experimental condition. Data are expressed as means ± SEM. Significance of the differences: *—*p* < 0.05 versus Co; Figure S2. Ubiquitylated protein levels in myotube cultures exposed for 24 and 48 h to OC, CM-C26 or TNF-α. The results are representative of three experiments, and each experimental condition is duplicated. Data are expressed as means ± SEM. Significance of the differences: *—*p* < 0.05 vs. Co; #—*p* < 0.05 vs. CM-C26. Figure S3. Markers of autophagy in myotube cultures exposed for 24 and 48 h to OC, CM-C26 or TNF-α. The results are representative of three experiments, and each experimental condition is duplicated. Data are expressed as means ± SEM. Significance of the differences: *—*p* < 0.05 vs. Co; #—*p* < 0.05 vs. CM-C26.
