# Supplementary material for: Oleocanthal Protects C2C12 Myotubes against the Pro-Catabolic and Anti-Myogenic Action of Stimuli Able to Induce Muscle Wasting In Vivo
Source: Nutrients. 2024 Apr 26;16(9):1302. doi: 10.3390/nu16091302 (PMC11085360; doi:10.3390/nu16091302)
Supplement: Supplementary file 1 [file nutrients-16-01302-s001.zip › nutrients-2970302-supplementary.pdf]

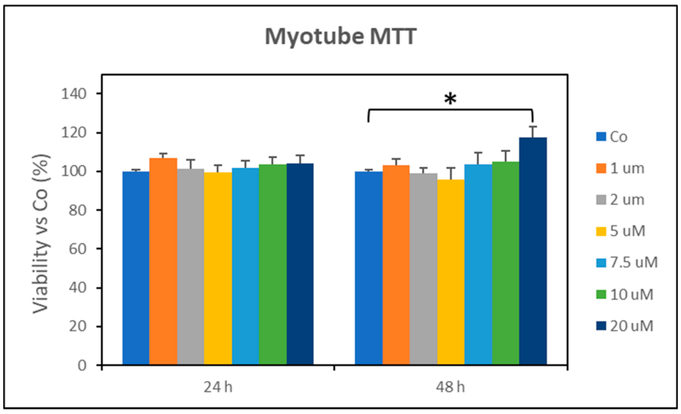

Figure S1. MTT assay on C2C12 myotube cultures exposed for 24 and 48 h to different OC concentrations. The results are representative of three experiments, with $n = 8$ for each experimental condition. Data are expressed as means ± SEM. Significance of the differences: *—$p < 0.05$ versus Co.

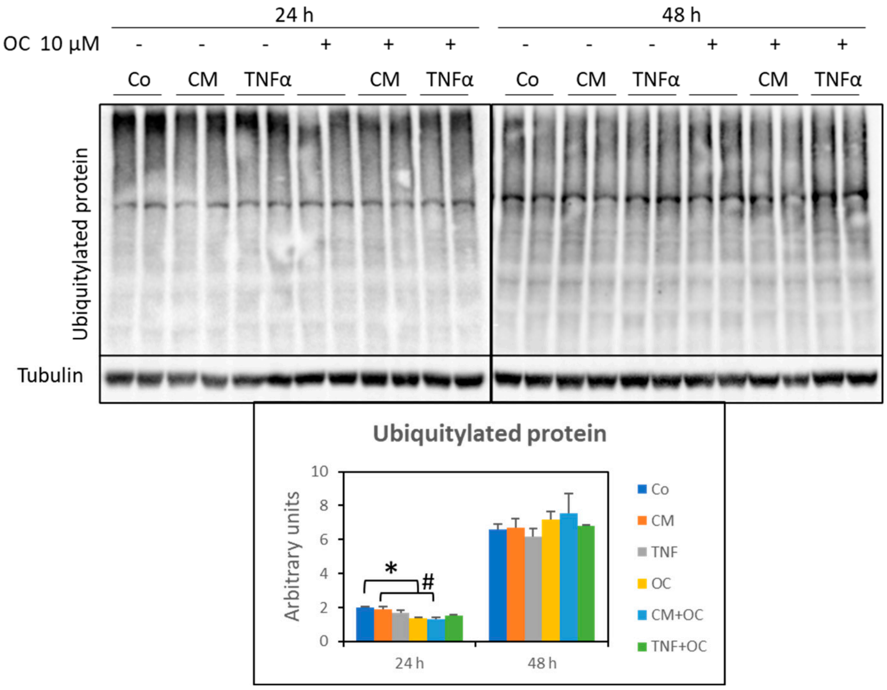

Figure S2. Ubiquitylated protein levels in myotube cultures exposed for 24 and 48 h to OC, CM-C26 or TNF-α. The results are representative of three experiments, and each experimental condition is duplicated. Data are expressed as means ± SEM. Significance of the differences: *—$p < 0.05$ vs. Co; #—$p < 0.05$ vs. CM-C26.

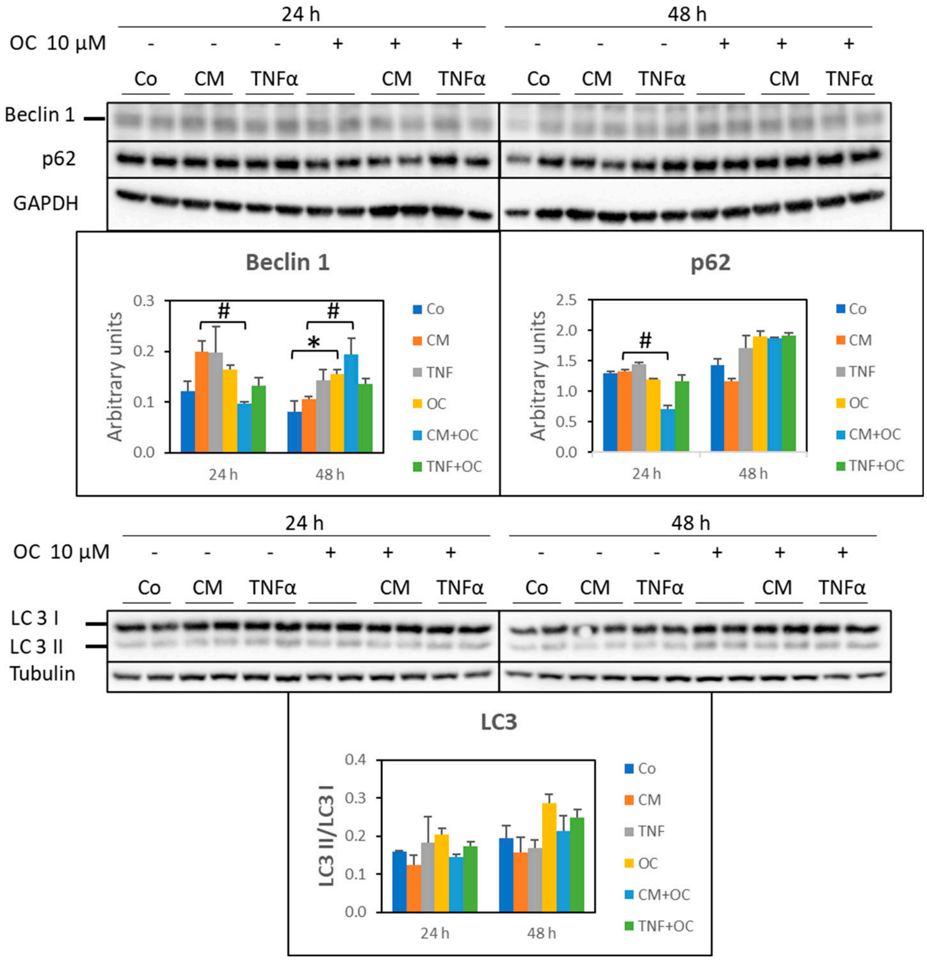

Figure S3. Markers of autophagy in myotube cultures exposed for 24 and 48 h to OC, CM-C26 or TNF-*α*. The results are representative of three experiments, and each experimental condition is duplicated. Data are expressed as means ± SEM. Significance of the differences: *—$p < 0.05$ vs. Co; #—$p < 0.05$ vs. CM-C26.
